# Is Sexual Size Dimorphism Inherent in the Scallop *Patinopecten yessoensis*?

**DOI:** 10.1155/2016/8653621

**Published:** 2016-05-12

**Authors:** Alla V. Silina

**Affiliations:** A. V. Zhirmunsky Institute of Marine Biology, Far East Branch of Russian Academy of Sciences, Vladivostok 690041, Russia

## Abstract

Studies on sexual size dimorphism in Pectinidae are limited. This work deals with the mobile long-lived scallop* Patinopecten yessoensis*, a common (fished and cultured) species in the subtidal benthos of the Sea of Japan. A previously developed method of age determination in* P. yessoensis* allowed me to compare the parameters of same aged males and females in scallop populations. The shell growth rates and sizes of both sexes were similar; therefore, it was only possible to visually identify the sex of live specimens during the breeding period (May-June). Statistical analyses showed female-biased dimorphism in the gonad weight for age groups that are >4 years old. Gonad weight (in the prespawning period) increased with age, until a threshold age was attained, which varied between populations; and then gonad weight remained virtually unchanged. The fecundity advantage hypothesis for* P. yessoensis* with group mating and external fertilization is at least partly realized by physiological mechanisms, which cause older females to have larger gonads than those of same aged males in the population in order to produce a larger brood. Gregarious settlement of this bivalve contributes to the reproductive success of the population so that the energetically costly ovaries may all be fertilized.

## 1. Introduction

The phenomenon referred to as sexual size dimorphism (SSD), in which males and females differ in their body size, is common in many animal taxa and is usually interpreted as the result of sexual selection [1, 2]. Cox with coauthors [3] suggested that SSD follows from two adaptive hypotheses: (1) the intrasexual selection hypothesis, by which the competition between males leads to selection for a larger body size, and (2) the fecundity advantage hypothesis, according to which natural selection drives females to have larger bodies in order to produce a larger brood size.

Reports about SSD in marine bivalves are rare.* Transennella* spp.,* Mytilus edulis*, and* Calyptogena gallardoi* are the known sexually dimorphic marine bivalves [4–6]. However, it is not clear whether the paucity of records of SSD reflects the fact that most bivalves are truly monomorphic or whether it is simply a consequence of the inherent difficulties in sexing bivalves by visual gonad inspection, particularly during nonbreeding periods. In addition, in natural populations, bivalve growth rates vary under different environments; therefore same-sized bivalves may have different ages, and this circumstance, in the absence of a method to determine individual age, hampers the comparison of results for different populations and cohorts.

Sexual size dimorphism is predicted to vary across mating systems [7]. Mating systems with males larger than females occur when males compete for female access or guard territories, while mating systems with group mating (many males participate in spawning events) tend to occur in species where females are the same size or larger than males [7]. To date, it is known that, at a given age, females are larger than males in* M. edulis* and* C. gallardoi*, both having group mating systems [5, 6].

A vast literature exists on all aspects of the ecology, development, and growth of the Japanese scallop* Patinopecten (Mizuhopecten) yessoensis* (Jay, 1856). However, no data on sexual size monomorphism or dimorphism have yet been reported for this species. The Japanese scallop is a group spawner; therefore, it is expected that either the species will have female-biased SSD or there will be no difference in size between the sexes.

We were able to investigate the sex ratio of each age cohort in a population of* P. yessoensis* as a method for ageing and determining shell height growth of each scallop through its lifetime has been devised [8, 9]. It uses the growth layers revealed in the external surface microsculpture of the upper valve. Winter and summer growth layers differ in their appearance and width. The scallop forms one broad elementary growth layer weekly during November–April and one narrow elementary growth layer daily during the rest of the year. The visible thickening of the narrowest layers (ring) occurs annually, in August. This allows both the age of each scallop, by counting the number of annual shell rings, and its individual growth rates, in retrospect by measuring the heights of the scallop shell from its apex to each annual ring, to be determined [8, 9]. The sex of the scallops may be determined visually during breeding periods [10–12]. Therefore,* P. yessoensis* is an appropriate species with which to investigate the life reproductive strategy of representative of the bivalve genus Pectinidae.

*Patinopecten yessoensis* is common to subtidal coasts of the Sea of Japan (East Sea) and the Okhotsk Sea. This scallop is a large-bodied (up to 220 mm in shell height), long-lived (lifetime of a maximum of 22 years), and mobile species [13]. Sexual maturity occurs at 2-3 years of age, depending on the habitat. It is dioecious, with a low incidence of hermaphrodites [10, 14]. Spawning occurs once a year; the spawning period can vary with environment but is usually limited to 1–1.5 months during the spring–summer [10–12, 15, 16]. Fertilization is external; the sperm and eggs are released into the sea water. Each female spawns ejecting (up to 10 times) portions of the eggs (≤10 millions in each portion) during 2-3 weeks. The males release the sperm up to 60 times during the spawning, and one such releasing lasts 45–50 sec [10]. Individual eggs are small, 50–80 *µ*m in diameter [12, 17].

The goal of this study was a comparison of shell parameters, growth patterns, and tissue weights of the same aged males and females of the Japanese scallop from different natural populations to investigate its SSD. In addition, I aimed to accurately compare the gonad weights of the same aged males and females to investigate the life reproductive strategy of the species.

## 2. Materials and Methods

**Figure 1:**
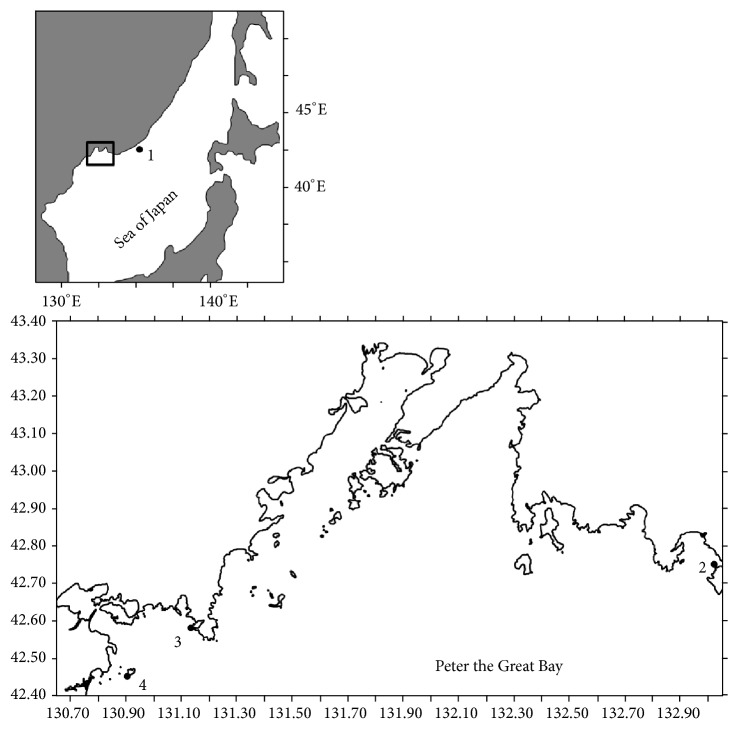
Sampling sites of scallop* Patinopecten yessoensis*: (1) Melkovodnaya Bay; (2) Ozero Vtoroe Bay; (3) Vityaz Bay; (4) Zapadnaya Bay of Furugalm Island. On both maps, black circles indicate the sites of scallop collection.

Japanese scallops from wild populations were collected by SCUBA divers at four sites in the northwestern Sea of Japan, mainly in Peter the Great Bay (Figure 1). The samples comprised 126, 197, 80, and 100 scallops from Melkovodnaya Bay, Ozero Vtoroe Bay, Vityaz Bay, and Zapadnaya Bay of Furugalm Island, respectively. Individuals were sampled just before or at the beginning of spawning, when their gonads were mature (May-June) and the sex of the scallops can be ascertained visually by noting the color of the gonad.* Patinopecten yessoensis* female gonad is pink, while the male gonad is creamy [10–12].

 In order to accurately discriminate between Japanese scallops based on their sex using morphometric characters, eight parameters were measured in specimens of known sex. Shell height (dorsoventral axis), length (anteroposterior axis), and width (lateral axis) were measured to the nearest 0.1 mm with vernier calipers. Total live weight with shell closed and total shell weight were determined with a Shimadzu electric scale to the nearest 0.1 g. Adductor muscle and gonad were separated from soft tissues and weighed with an electric scale. The remains of internal soft tissues were weighed, too.

The age of each scallop was determined by counting the number of annual shell rings [8, 9]. Shell heights every year over the scallop's lifetime were determined retrospectively from the growth layers revealed in the microsculpture of the external surface of the upper valve and measured from scallop's apex to each annual ring to the nearest 0.1 mm with vernier calipers. The growth rates of the same aged males and females were compared in each population. Also, mean growth rates of the scallops were compared between the populations.

The mean ± SEM (standard error of the mean) values were obtained for the morphometric characters of males and females in each population. A* t*-test was applied to reveal differences between the mean values of the same aged male and female parameters in the populations. Prior to statistical analysis, all data were tested for normality of variance among the different groups by using a Kolmogorov-Smirnov test. In wild populations, the numbers of individuals at each given age are often not numerous due to both interannual irregular replenishment and natural mortality increasing with the age of scallop; therefore, for comparative statistical analyses, usually the representative samples of males and females were chosen (the number of males ≥5 ind. and the number of females ≥5 ind.).

## 3. Results and Discussion

**Figure 2:**
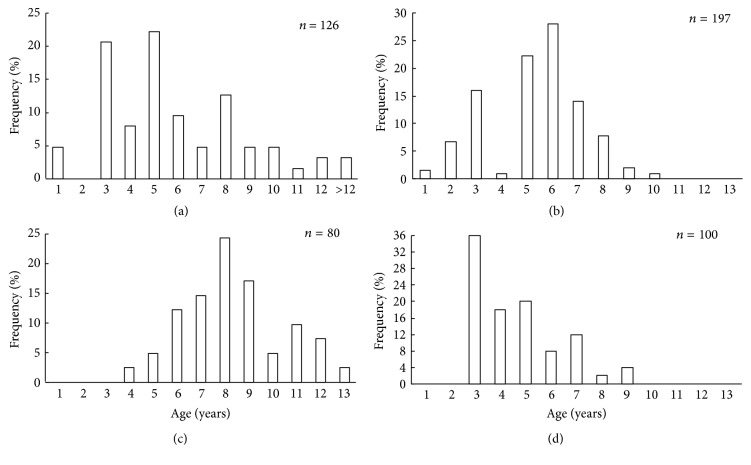
*Patinopecten yessoensis.* Age structures of the scallop populations from (a) Melkovodnaya Bay, (b) Ozero Vtoroe Bay, (c) Vityaz Bay, and (d) Zapadnaya Bay of Furugalm Island.* n* is sample size.

It was found that scallop samples from Melkovodnaya Bay, Ozero Vtoroe Bay, Vityaz Bay, and Zapadnaya Bay of Furugalm Island consisted of 1–14-, 1–10-, 4–13-, and 3–9-year-old individuals (Figure 2). Sex ratios differed among the scallop populations, ranging between 0.90 : 1 and 0.96 : 1 (males : females), and the mean sex ratio was 0.93 ± 0.01 (SD = 0.03) for four populations. The general sex ratio in the populations did not differ considerably from the expected Fisher 1 : 1 ratio for dioecious species, with a slight female-biased sex ratio overall. The above agrees with that reported for this scallop species; for example, Bregman [14] has found in general a 1 : 1 sex ratio for wild Japanese scallop populations. Intraspecific variation in the sex ratio equips the Bivalvia for the total range of aquatic habitats [18]. The frequency of occurrence of hermaphrodites was low, one or two per hundred. Usually, hermaphroditic gonads were creamy with small pink dots. Only a single specimen had a pink female gonad with creamy dots. The low frequency of hermaphrodite occurrence agrees with previously obtained data for studied scallop species [14].

**Figure 3:**
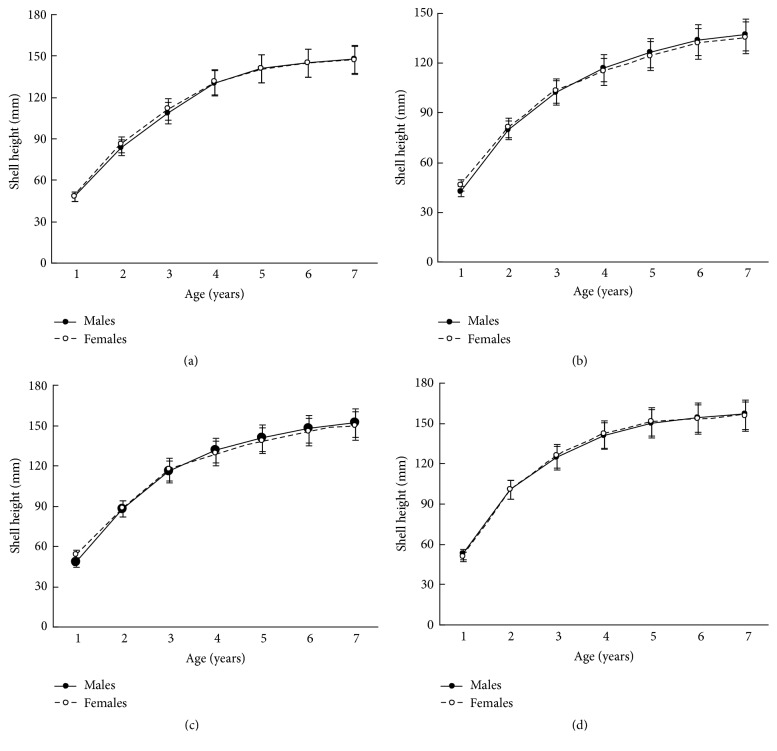
*Patinopecten yessoensis*. Shell height growth of 7-year-old males and females from (a) Melkovodnaya Bay, (b) Ozero Vtoroe Bay, (c) Vityaz Bay, and (d) Zapadnaya Bay of Furugalm Island. Data are mean ± SE.

The size and weight parameters were statistically compared between the same aged males and females. The shell size parameters (height, length, and width) of the same aged scallops were not sexually dimorphic (Tables 1 [Table tab2] [Table tab3]–4). The shell height growths of the same aged scallops of both genders were statistically equal throughout all their lifetimes (Figure 3).

The mean total weights of the same aged males and females were practically equal for young individuals or exhibited male-biased dimorphism for ≥5-year-olds (Tables 1 [Table tab2] [Table tab3]–4). A closer examination of weight revealed that it was scallop shell weight that was driving this male-biased dimorphism in total weight and to a lesser extent adductor muscle weight (Tables 1 [Table tab2] [Table tab3]–4). A similar tendency was found for the scallop shell weight (Tables 1 [Table tab2] [Table tab3]–4). Sexual dimorphism was not evident during the scallop life in such parameter as adductor muscle weight, though it was found for some ages that the adductor muscles of males were larger than those of females (Tables 1 [Table tab2] [Table tab3]–4). The shell weight of the middle- and old-aged males is larger than that of females possibly due to a greater frequency and duration of a gamete released by males (60 times per spawning) than females (≤10 times). During ejection of gametes, the scallop strongly claps its valves, and the shell edge breaks off. Restoration of the edge requires the deposition of additional calcite layers on the inner surface of the shell [8]; therefore, considerable edge reconstruction makes the male's shell thicker and heavier than a female's. This is the main cause of the larger total weight of middle- and old-aged males compared to females, as shell weight is usually half of the total weight. The greater efforts made by males in spawning may also influence the strengthening of their adductor muscles.

**Figure 4:**
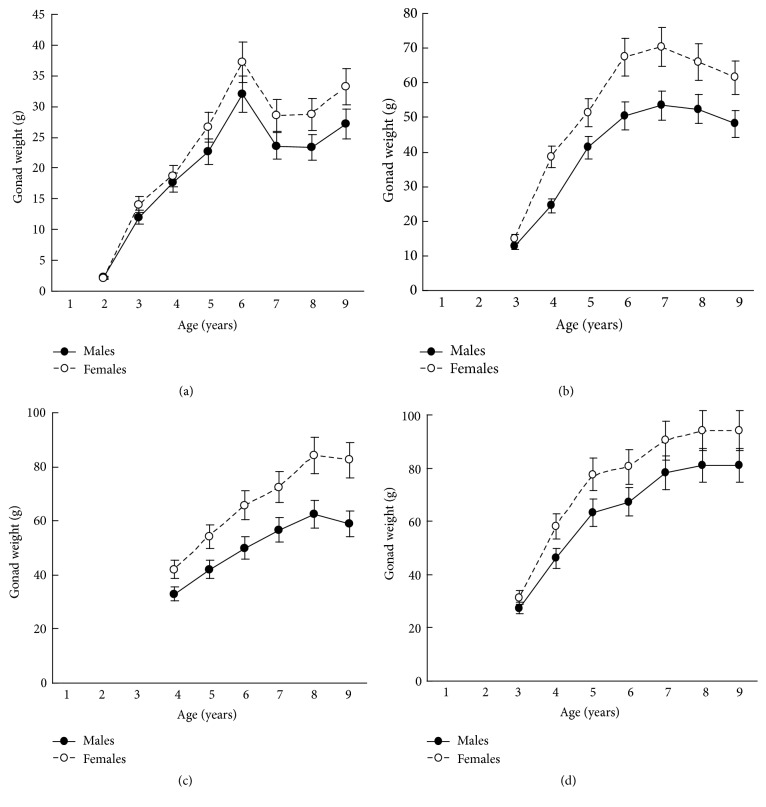
*Patinopecten yessoensis*. Gonad weight growth of the males and females in the studied sites: (a) Ozero Vtoroe Bay; (b) Melkovodnaya Bay; (c) Vityaz Bay; (d) Zapadnaya Bay of Furugalm Island. Data are mean ± SE.

**Figure 5:**
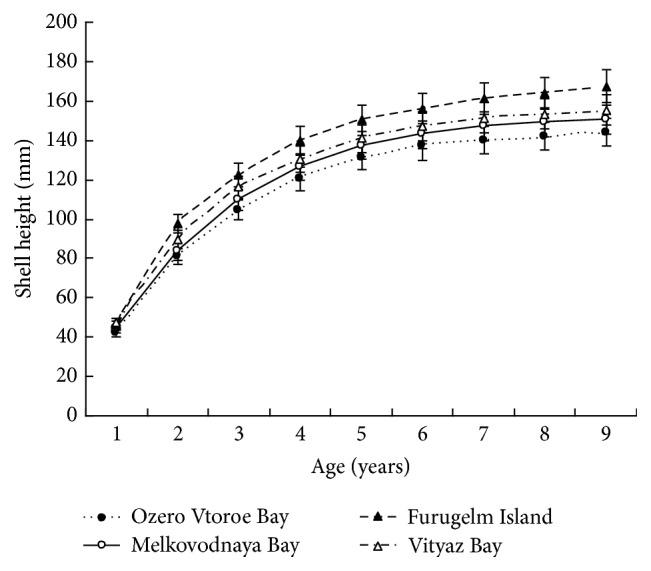
*Patinopecten yessoensis*. Growth of the scallops in the studied sites. Data are mean ± SE.

The results in terms of reproductive effort were illustrated by gonad weight. The mean estimated gonad weights of both males and females increased with age (Tables 1 [Table tab2] [Table tab3]–4, Figure 4). Gonad weights increased until a threshold age was attained, which varied between the populations; and later the gonad weight was, on the whole, virtually unchanged or somewhat decreased for the oldest age classes (Figure 4). For instance, in the populations inhabiting Ozero Vtoroe Bay, Melkovodnaya Bay, Vityaz Bay, and Zapadnaya Bay of Furugelm Island, the threshold ages were found for individuals of 6, 7, 8, and 8 years of age, respectively (Figure 4). The increase in the threshold age between these sites was in accordance with the increase in the growth rates of the scallops at the sites (Figures 4 and 5). The shell heights were significantly (*t*-test,* P* < 0.01) higher at Zapadnaya Bay of Furugelm Island than at Ozero Vtoroe Bay for all scallop ages (Figure 5). It is known that growth is an integrative index of organism condition in a certain environment. Actually, the habitats near Furugelm Island, in Vityaz Bay and Melkovodnaya Bay, are more optimal for Japanese scallops than those in Ozero Vtoroe Bay [13]. At the latter bay, the bottom sediments have a high mud concentration and it is known that the Japanese scallop is sensitive to suspended mud [10]. Furthermore, Ozero Vtoroe Bay is greatly sheltered; these conditions make water exchange weak. Therefore, the water salinity and oxygen concentration in this bay are too low in summer, and the water temperature is too high for the scallop [10]. The habitat in Zapadnaya Bay of Furugelm Island is most optimal for Japanese scallops. It is obvious that favorable environmental conditions provide sufficient resources for both somatic and reproductive growth of older mollusks. In the middle- and old-aged ontogenesis stages of scallop's development, the differences in the gonad weight between genders were usually statistically significant (*t*-test,* P* < 0.01–0.05) with the highest values occurring in female scallops (Figure 4).

Thus, statistical analyses revealed differences in scallop gonad weight between genders. The mean gonad weights showed female-biased dimorphism for middle- and old-age classes of scallops. Obtained data are in accordance with the fecundity advantage hypothesis of Cox and coauthors [3], which suggests that natural selection drives females to have larger bodies (gonads, for Japanese scallop) in order to produce a larger brood size. Female scallops invest their energy in increasing gonad weight, whereas males have to spend their energy on shell reconstruction (increase of shell weight) and strengthening of their adductor muscle.

## 4. Conclusion

The Japanese scallop is long-lived (maximum up to 22 years) but becomes mature early, at the age of 2-3 years. Early energy is therefore put into gonadal development. In such a case, it is expected that an animal can interrupt or reduce their somatic growth [19, 20]. Both males and females show somatic growth during all their lives. Also, marked differences between age classes are found in reproductive investment, with young having a lower reproductive output. The gonad weight increases until the scallop attains a threshold age, which varies between the populations. Although the rate of change in fecundity apparently declines in old scallops, these aged individuals continue to have large gonads.

As the Japanese scallop is a group spawner, it is expected that the species has a female-biased SSD or both sexes have the same size. Actually, pronounced SSD of* P. yessoensis* is not found between the same aged individuals of different genders. The shell growth rates and size parameters of both scallop genders of the same age do not differ statistically. Due to insignificant difference between the mean shell sizes of male and female scallops, it is impossibleto visually identify the sex of live specimens during nonbreeding periods.

The differences between the same aged genders become significant for older aged scallops for such parameters as gonad weight (female-biased dimorphism for middle and old age classes, ≥5 years old). The fecundity advantage hypothesis for the Japanese scallop with group mating is realized by physiological mechanisms that ensure larger gonads in senior females compared to males in a population in order to produce a larger clutch. Gregarious habitation of this bivalve aids population reproductive success. With males in the vicinity, the probability rises that the energetically costly ovaries will all be fertilized.

## Figures and Tables

**Table 1:** *Patinopecten yessoensis*. Wet weight and size parameters of the same aged males and females from the population inhabiting Melkovodnaya Bay. *∗* indicates significant differences between parameters at a level *P* < 0.05 (*t*-test). — indicates that data were not compared. Bold marks higher values. *n* is sample size.

Parameters, g	Males	Females	*P*
3-year-olds	*n* = 20	*n* = 6	

Total weight	161.9 ± 10.0	159.2 ± 7.7	0.3652
Adductor muscle weight	20.2 ± 1.9	18.4 ± 2.1	0.2650
Shell weight	72.1 ± 5.6	70.8 ± 5.6	0.2542
Remains of soft tissues without gonads	22.1 ± 2.1	18.6 ± 3.0	0.0821
Shell height	106.6 ± 3.1	104.0 ± 3.8	0.2864
Shell length	110.0 ± 4.3	107.8 ± 4.1	0.3246
Shell width	27.5 ± 0.6	25.6 ± 1.2	0.0620

4-year-olds	*n* = 6	*n* = 4	

Total weight	294.7 ± 8.5	285.8 ± 10.5	0.3548
Adductor muscle weight	40.4 ± 1.6	37.7 ± 3.0	0.3673
Shell weight	129.0 ± 5.2	119.8 ± 5.6	0.0734
Remains of soft tissues without gonads	32.5 ± 2.0	30.3 ± 2.3	0.1035
Shell height	123.7 ± 2.9	122.1 ± 3.2	0.4891
Shell length	129.9 ± 3.4	128.5 ± 4.0	0.3468
Shell width	32.5 ± 0.9	31.3 ± 1.1	0.0773

5-year-olds	*n* = 16	*n* = 12	

Total weight	404.8 ± 19.0	402.7 ± 20.2	0.5331
Adductor muscle weight	55.9 ± 1.8^*∗*^	50.9 ± 3.1^*∗*^	0.0425
Shell weight	199.7 ± 11.3	188.0 ± 11.2	0.1483
Remains of soft tissues without gonads	52.2 ± 2.4	51.7 ± 2.9	0.2115
Shell height	138.2 ± 1.7	137.8 ± 2.7	0.5857
Shell length	146.8 ± 2.1	146.0 ± 2.9	0.7625
Shell width	38.5 ± 0.8	38.0 ± 1.4	0.0598

6-year-olds	*n* = 6	*n* = 6	

Total weight	505.1 ± 10.7^*∗*^	445.3 ± 13.5^*∗*^	0.0353
Adductor muscle weight	72.0 ± 4.4	65.3 ± 3.1	0.0621
Shell weight	244.6 ± 9.5^*∗*^	213.8 ± 8.4^*∗*^	0.0432
Remains of soft tissues without gonads	65.9 ± 6.1	62.4 ± 5.5	0.0958
Shell height	146.4 ± 4.8	147.0 ± 4.8	0.5697
Shell length	159.3 ± 5.5	160.5 ± 4.9	0.4627
Shell width	41.3 ± 0.9	42.0 ± 0.9	0.1068

7-year-olds	*n* = 3	*n* = 3	

Total weight	527.3 ± 20.1	489.1 ± 14.5	—
Adductor muscle weight	72.4 ± 4.1	70.1 ± 3.4	—
Shell weight	264.4 ± 10.6	237.6 ± 9.5	—
Remains of soft tissues without gonads	70.7 ± 2.8	67.4 ± 2.8	—
Shell height	147.5 ± 4.0	146.7 ± 4.7	—
Shell length	162.5 ± 7.7	161.7 ± 5.2	—
Shell width	43.8 ± 1.5	43.2 ± 1.9	—

8-year-olds	*n* = 6	*n* = 10	

Total weight	592.4 ± 14.4^*∗*^	551.2 ± 11.7^*∗*^	0.0413
Adductor muscle weight	75.1 ± 3.9	74.0 ± 3.6	0.3422
Shell weight	300.5 ± 12.9^*∗*^	271.3 ± 10.1^*∗*^	0.0480
Remains of soft tissues without gonads	63.7 ± 2.0	65.3 ± 1.2	0.1653
Shell height	148.9 ± 4.3	150.4 ± 2.6	0.4897
Shell length	160.0 ± 5.1	163.4 ± 5.0	0.3685
Shell width	44.8 ± 1.6	44.2 ± 0.4	0.3597

**Table 2 tab2:** *Patinopecten yessoensis*. Wet weight and size parameters of the same aged males and females from the population inhabiting Ozero Vtoroe Bay. *∗* indicates significant differences between parameters at a level *P* < 0.05 (*t*-test). — indicates that data were not compared. Bold marks higher values. *n* is sample size.

Parameters, g	Males	Females	*P*

2-year-olds	*n* = 10	*n* = 2	

Total weight	63.7 ± 4.8	63.9 ± 8.1	—
Adductor muscle weight	8.7 ± 0.5	9.6 ± 2.5	—
Shell weight	31.4 ± 2.2	30.5 ± 2.5	—
Remains of soft tissues without gonads	9.5 ± 0.7	9.9 ± 0.5	—
Shell height	78.2 ± 1.8	78.5 ± 3.5	—
Shell length	82.4 ± 2.0	83.8 ± 2.3	—
Shell width	19.3 ± 0.7	19.0 ± 1.7	—

3-year-olds	*n* = 22	*n* = 11	

Total weight	163.1 ± 8.5	167.4 ± 8.7	0.3641
Adductor muscle weight	28.5 ± 1.4	28.0 ± 1.9	0.6659
Shell weight	73.8 ± 4.1	74.5 ± 3.6	0.4440
Remains of soft tissues without gonads	22.4 ± 1.1	24.0 ± 1.1	0.1081
Shell height	110.6 ± 1.7	114.2 ± 1.5	0.0869
Shell length	113.5 ± 1.9	116.9 ± 1.9	0.1046
Shell width	28.0 ± 0.7	29.7 ± 0.7	0.0823

4-year-olds	*n* = 3	*n* = 2	

Total weight	297.9 ± 8.5	266.6 ± 10.8	—
Adductor muscle weight	46.7 ± 1.4	32.0 ± 2.6	—
Shell weight	129.0 ± 5.2	123.8 ± 4.6	—
Remains of soft tissues without gonads	32.4 ± 1.1	30.4 ± 1.9	—
Shell height	132.6 ± 1.9	130.2 ± 2.5	—
Shell length	136.1 ± 2.0	132.5 ± 2.7	—
Shell width	34.1 ± 1.2	34.0 ± 1.9	—

5-year-olds	*n* = 18	*n* = 24	

Total weight	377.6 ± 18.1^*∗*^	339.4 ± 11.7^*∗*^	0.0433
Adductor muscle weight	58.3 ± 2.9^*∗*^	52.4 ± 1.7^*∗*^	0.0421
Shell weight	168.4 ± 8.9^*∗*^	150.1 ± 6.0^*∗*^	0.0489
Remains of soft tissues without gonads	48.7 ± 2.1	45.6 ± 1.5	0.0651
Shell height	134.8 ± 1.9	132.5 ± 1.6	0.1887
Shell length	145.5 ± 2.9	141.8 ± 1.8	0.1625
Shell width	38.0 ± 0.9	36.6 ± 0.5	0.0598

6-year-olds	*n* = 22	*n* = 27	

Total weight	416.6 ± 12.5	403.8 ± 12.5	0.2504
Adductor muscle weight	64.7 ± 2.2	63.6 ± 2.4	0.3693
Shell weight	181.0 ± 6.6	171.8 ± 6.0	0.1584
Remains of soft tissues without gonads	53.9 ± 1.5	51.6 ± 1.7	0.1589
Shell height	139.3 ± 1.5	137.5 ± 1.6	0.4153
Shell length	150.6 ± 2.0	149.6 ± 1.8	0.7420
Shell width	39.7 ± 0.5	39.1 ± 0.6	0.4687

7-year-olds	*n* = 10	*n* = 18	

Total weight	379.8 ± 17.3	366.0 ± 16.5	0.2923
Adductor muscle weight	55.2 ± 2.8	55.7 ± 3.1	0.4581
Shell weight	163.2 ± 9.4	158.8 ± 8.4	0.3353
Remains of soft tissues without gonads	50.3 ± 2.6	46.1 ± 2.1	0.1190
Shell height	136.7 ± 2.1	134.7 ± 2.2	0.5567
Shell length	147.7 ± 2.4	146.3 ± 2.8	0.7270
Shell width	37.2 ± 0.7	36.4 ± 0.6	0.4467

8-year-olds	*n* = 7	*n* = 12	

Total weight	438.1 ± 18.1^*∗*^	407.0 ± 11.7^*∗*^	0.0360
Adductor muscle weight	68.3 ± 2.9^*∗*^	59.0 ± 1.7^*∗*^	0.0191
Shell weight	200.1 ± 8.9^*∗*^	172.1 ± 6.0^*∗*^	0.0097
Remains of soft tissues without gonads	55.1 ± 3.4	51.8 ± 2.8	0.2276
Shell height	140.6 ± 3.3	137.8 ± 2.1	0.5013
Shell length	152.7 ± 2.9	150.9 ± 2.4	0.6371
Shell width	39.1 ± 0.8	39.8 ± 0.5	0.5250

**Table 3 tab3:** *Patinopecten yessoensis*. Wet weight and size parameters of the same aged males and females from the population inhabiting Vityaz Bay. *∗* indicates significant differences between parameters at a level *P* < 0.05 (*t*-test). Bold marks higher values. *n* is sample size.

Parameters, g	Males	Females	*P*
6-year-olds	*n* = 6	*n* = 4	

Total weight	411.1 ± 8.5^*∗*^	369.4 ± 11.7^*∗*^	0.0343
Adductor muscle weight	60.3 ± 2.9	56.4 ± 1.7	0.0732
Shell weight	185.3 ± 5.2^*∗*^	169.8 ± 6.0^*∗*^	0.0445
Remains of soft tissues without gonads	59.5 ± 3.3	58.7 ± 3.2	0.0951
Shell height	142.7 ± 4.3	142.5 ± 4.0	0.8176
Shell length	152.5 ± 4.3	152.3 ± 3.9	0.8627
Shell width	39.8 ± 1.0	38.7 ± 0.8	0.0989

7-year-olds	*n* = 7	*n* = 5	

Total weight	469.5 ± 12.5^*∗*^	429.3 ± 17.5^*∗*^	0.0463
Adductor muscle weight	69.5 ± 2.2	64.4 ± 4.5	0.0627
Shell weight	220.0 ± 6.6^*∗*^	193.9 ± 7.2^*∗*^	0.0382
Remains of soft tissues without gonads	68.0 ± 3.9	65.9 ± 4.0	0.0678
Shell height	151.5 ± 2.8	150.3 ± 1.1	0.6897
Shell length	161.1 ± 2.8	158.5 ± 3.6	0.3625
Shell width	42.1 ± 1.6	41.7 ± 1.4	0.0998

8-year-olds	*n* = 11	*n* = 9	

Total weight	507.1 ± 18.9^*∗*^	459.5 ± 16.2	0.0373
Adductor muscle weight	74.0 ± 1.5	68.8 ± 4.6	0.0727
Shell weight	236.2 ± 5.7^*∗*^	211.2 ± 5.7^*∗*^	0.0382
Remains of soft tissues without gonads	75.5 ± 5.2	73.6 ± 5.2	0.0853
Shell height	155.5 ± 4.4	153.8 ± 2.9	0.4837
Shell length	164.3 ± 4.6	162.2 ± 3.3	0.2685
Shell width	43.3 ± 0.8	42.4 ± 0.8	0.0989

9-year-olds	*n* = 6	*n* = 8	

Total weight	530.5 ± 18.8	510.7 ± 14.6	0.1273
Adductor muscle weight	76.5 ± 3.2^*∗*^	68.5 ± 4.5^*∗*^	0.0491
Shell weight	240.2 ± 12.4	233.4 ± 10.9	0.2482
Remains of soft tissues without gonads	83.3 ± 8.4	82.2 ± 6.6	0.1659
Shell height	158.7 ± 1.3	158.5 ± 2.3	0.8184
Shell length	168.0 ± 2.6	168.3 ± 3.8	0.8020
Shell width	42.2 ± 0.9	43.1 ± 0.6	0.0856

**Table 4:** *Patinopecten yessoensis*. Wet weight and size parameters of the same aged males and females from the population inhabiting Zapadnaya Bay of Furugalm Island. *∗* indicates significant differences between parameters at a level *P* < 0.05 (*t*-test). — indicates that data were not compared. Bold marks higher values. *n* is sample size.

Parameters, g	Males	Females	*P*
3-year-olds	*n* = 24	*n* = 12	

Total weight	201.5 ± 8.5	187.4 ± 8.7	0.1653
Adductor muscle weight	37.5 ± 1.4	34.6 ± 1.9	0.1701
Shell weight	94.3 ± 2.1	89.0 ± 2.5	0.0654
Remains of soft tissues without gonads	34.4 ± 2.1	32.4 ± 2.7	0.0819
Shell height	121.3 ± 3.0	119.0 ± 3.5	0.4697
Shell length	123.0 ± 2.9	122.8 ± 3.4	0.7046
Shell width	17.9 ± 0.6	17.4 ± 0.6	0.1831

4-year-olds	*n* = 4	*n* = 14	

Total weight	318.9 ± 9.5^*∗*^	286.6 ± 8.8^*∗*^	0.0373
Adductor muscle weight	55.2 ± 2.8	55.7 ± 3.1	0.4232
Shell weight	150.6 ± 5.2^*∗*^	140.1 ± 3.1^*∗*^	0.0412
Remains of soft tissues without gonads	50.5 ± 4.3	47.5 ± 2.2	0.0689
Shell height	139.5 ± 5.2	136.1 ± 3.5	0.3858
Shell length	143.5 ± 5.4	142.7 ± 3.6	0.7626
Shell width	23.4 ± 1.5	23.0 ± 1.0	0.1591

5-year-olds	*n* = 12	*n* = 8	

Total weight	387.8 ± 17.3^*∗*^	350.4 ± 11.7^*∗*^	0.0393
Adductor muscle weight	61.5 ± 2.4	58.1 ± 1.6	0.0822
Shell weight	183.2 ± 9.4^*∗*^	158.8 ± 8.4^*∗*^	0.0311
Remains of soft tissues without gonads	68.8 ± 2.2	65.1 ± 3.1	0.1621
Shell height	150.8 ± 3.8	148.3 ± 4.1	0.6837
Shell length	156.2 ± 4.0	155.3 ± 4.5	0.5645
Shell width	29.2 ± 0.8	27.0 ± 1.1	0.0582

6-year-olds	*n* = 6	*n* = 2	

Total weight	404.2 ± 18.1	366.0 ± 16.5	—
Adductor muscle weight	64.7 ± 2.2	63.6 ± 2.4	—
Shell weight	196.3 ± 6.9	162.1 ± 6.0^*∗*^	—
Remains of soft tissues without gonads	73.3 ± 4.3	69.6 ± 3.0	—
Shell height	158.0 ± 3.9	155.7 ± 4.0	—
Shell length	161.0 ± 3.6	157.3 ± 4.0	—
Shell width	32.3 ± 0.8	31.3 ± 1.3	—

7-year-olds	*n* = 6	*n* = 6	

Total weight	482.1 ± 12.5^*∗*^	433.8 ± 12.5^*∗*^	0.0235
Adductor muscle weight	70.3 ± 2.9	66.4 ± 1.7	0.0498
Shell weight	231.0 ± 6.6^*∗*^	191.8 ± 6.0^*∗*^	0.0293
Remains of soft tissues without gonads	73.3 ± 3.8	71.5 ± 4.0	0.1049
Shell height	161.3 ± 4.0	160.0 ± 3.6	0.4896
Shell length	165.3 ± 3.9	165.7 ± 2.9	0.7162
Shell width	30.3 ± 0.9	29.3 ± 1.1	0.0609
